# Integrating databases for spatial analysis of parasite-host associations and the novel Brazilian dataset

**DOI:** 10.1038/s41597-023-02636-8

**Published:** 2023-11-02

**Authors:** Gabriella L. T. Cruz, Gisele R. Winck, Paulo S. D’Andrea, Eduardo Krempser, Mariana M. Vidal, Cecilia S. Andreazzi

**Affiliations:** 1 Laboratório de Biologia e Parasitologia de Mamíferos Silvestres Reservatórios (LABPMR), Instituto Oswaldo Cruz, Fundação Oswaldo Cruz (Fiocruz), Rio de Janeiro, RJ Brazil; 2 Programa de Pós-graduação em Biodiversidade e Saúde, Instituto Oswaldo Cruz (IOC), Fundação Oswaldo Cruz (Fiocruz), Rio de Janeiro, RJ Brazil; 3 Pró-Reitoria de Pós-Graduação, Pesquisa e Inovação (PROPGPI), Universidade Federal do Estado do Rio de Janeiro (Unirio), Rio de Janeiro, RJ Brazil; 4 Plataforma Institucional Biodiversidade e Saúde Silvestre (PIBSS), Fundação Oswaldo Cruz (Fiocruz), Rio de Janeiro, RJ Brazil; 5 International Platform for Science, Technology and Innovation in Health (PICTIS), Ílhavo, Portugal; 6 Departamento de Biodiversidad, Ecología y Evolución, Universidad Complutense de Madrid, Madrid, Spain

**Keywords:** Biogeography, Infectious diseases, Ecological epidemiology

## Abstract

Incomplete information on parasites, their associated hosts, and their precise geographical location hampers the ability to predict disease emergence in Brazil, a continental-sized country characterised by significant regional disparities. Here, we demonstrate how the NCBI Nucleotide and GBIF databases can be used as complementary databases to study spatially georeferenced parasite-host associations. We also provide a comprehensive dataset of parasites associated with mammal species that occur in Brazil, the Brazilian Mammal Parasite Occurrence Data (BMPO). This dataset integrates wild mammal species’ morphological and life-history traits, zoonotic parasite status, and zoonotic microparasite transmission modes. Through meta-networks, comprising interconnected host species linked by shared zoonotic microparasites, we elucidate patterns of zoonotic microparasite dissemination. This approach contributes to wild animal and zoonoses surveillance, identifying and targeting host species accountable for disproportionate levels of parasite sharing within distinct biomes. Moreover, our novel dataset contributes to the refinement of models concerning disease emergence and parasite distribution among host species.

## Introduction

Parasite diversity, their associated hosts, and geographic distribution address many current scientific and social challenges, ranging from conservation issues to public health^1–3^. However, to properly understand parasite-host associations, their spatial dynamics, and public health implications, we need to grasp the spatial heterogeneity of these interactions and interdependence across taxa. Furthermore, to identify parasite transmission cycles and produce accurate predictive models on zoonotic disease emergence risks, the relative contributions of environmental and socioeconomic factors in driving species interactions at fine-scale spatial variations should also be understood (*e.g*., Márquez-Velásquez *et al*.^4^, Albery *et al*.^5^). Yet, efforts to include parasite-host association data in more complex evaluations are hampered mainly by incomplete parasite information, such as associated hosts and precise location on a fine geographic scale.

Global databases play a critical role in understanding zoonotic disease emergence risks. For instance, advances in knowledge concerning ecological patterns in parasite-sharing networks and predictions on potential new hosts for bat-betacoronavirus associations^6^, and for different mammal taxa and pathogens^7^, have employed large volumes of data from the NCBI Nucleotide database. NCBI Nucleotide is a public nucleic acid sequence database maintained by the National Center for Biotechnology Information (NCBI). On the other hand, the Global Biodiversity Information Facility (GBIF) is a global biodiversity occurrence repository that we believe can be used as a complementary source for NCBI Nucleotide data, since biodiversity studies applying or developing spatial models mostly use distribution data from GBIF (*e.g*., Staniczenko *et al*.^8^, Dallas *et al*.^9^, Redding *et al*.^10^). Even after Astorga *et al*.^11^ point out GBIF as a repository with potential use in studies of infectious diseases, as it contributes to aggregated and standardised data, it is necessary to review the quality of the data added to this repository regarding parasites and hosts.

Efforts are underway to understand the spatial distribution of parasite richness^2,12,13^ and pathogen-host range (see Shaw *et al*.^14^ for an overview) on a global scale. Some of these studies have resulted in global parasite-host association evaluation maps at the country level, revealing Brazil as a parasite diversity hotspot. However, Brazilian territory comprises a little over 8.5 million km^2^ ^15^ and includes six very different terrestrial biomes (Amazon Rainforest, Atlantic Rainforest, Caatinga, Cerrado, Pampa, and Pantanal) with their respective main ecotones (rainforests, dry forests, savannah, grasslands, and wetlands). Moreover, administrative territories comprise 5,570 municipalities, whose spatial organisations reflect the country’s historical occupation, with over 84% of the human population inhabiting coastal areas in the Northeastern, Southeastern, and Southern regions, according to according to the latest complete census data published^16^. Considering the environmental and population variations across this expansive country, in-depth analyses are crucial, as several of the estimated associations verified to date are potentially zoonotic^17,18^.

The goals of this study are twofold. Firstly, we argue that the two aforementioned open-access databases, NCBI Nucleotide and GBIF, hold complementary and important information to enhance disease ecology predictive models. Using Brazil as a study case, we exemplify how improving species association data quality aids model applicability, enabling robust predictions for large countries by downscaling geographic scales and including reliable parasite-host associations. Secondly, despite the importance of collecting data and extracting information to identify and predict risks, there are limited efforts to compile comprehensive datasets concerning the full set of parasites of free-ranging wild mammals in Brazil (*e.g*., Chame *et al*.^19^). Also, it is fair to assume that parasites, host richness, or their associations vary across the megadiverse and heterogeneous Brazilian territory. Therefore, we provide an extensive parasite-mammal association dataset at a fine geographic scale, the Brazilian Mammal Parasite Occurrence Data (BMPO), aiming at a comprehensive view of available information on parasitic associations throughout Brazil. This information was sourced from well-established datasets specifically tailored for parasite studies, the Enhanced Infectious Diseases database (EID2; Wardeh *et al*.^20^), and the Global Mammal Parasite Database v.2.0 (GMPD2; Stephens *et al*.^21^), and literature. We also provide information on morphological and life-history traits of indigenous mammal species, zoonotic parasite status, and the transmission modes of zoonotic microparasites.

Here, the term ‘parasite’ encompasses both macroparasites (arthropods and helminths) and microparasites (bacteria, fungi, protozoa, and viruses). Zoonotic microparasites denote microscopic pathogenic organisms with the dual capability of infecting and causing diseases in both animals and humans. Moreover, we posit that parasite-host associations imply the potential presence of one parasite within or on a host species, acknowledging that not all these associations represent parasite-host interactions. In this regard, through meta-networks, comprising interconnected host species linked by shared zoonotic microparasites, we elucidate patterns of zoonotic microparasite dissemination.

## Results

### What information on zoonotic microparasites is available in commonly used databases?

We found that many zoonotic microparasites are underrepresented in the EID2 and GMPD2 databases (Table 1) compared to the GBIF, NCBI Nucleotide databases, and our new dataset (BMPO). We also detected incomplete associated host/source data in the GBIF database. However, the GBIF data have a higher level of detail regarding the geographic location of the zoonotic microparasites (almost 100% of the data). Concerning NCBI Nucleotide data, only 30.7% of the analysed sequence records of the zoonotic microparasites contained geographical coordinates (N = 189,839, from a total of 618,360) (Supplementary Fig. S1). The majority of the records (70.3%) had humans as hosts (151 microparasites), and only 2% (1,257 sequences, 62 microparasites) had wild mammals as hosts (102 genera, 167 species). *Klebsiella pneumoniae* (N = 145,718) and *Escherichia coli* (N = 93,055) were the zoonotic microparasites with the highest number of recorded associations with host data.

**Table 1:** Summary of the different datasets used in this study, indicating the number of unique records (*i.e*., records on zoonotic microparasite species in Brazil) available at the NCBI Nucleotide database, the Enhanced Infectious Diseases (EID2) database, the Global Mammal Parasite v.2.0 (GMPD2) database, the Global Biodiversity Information Facility (GBIF) database, and records to free-ranging wild mammal species provided by our new dataset, the Brazilian Mammal Parasite Occurrence Data (BMPO).

Database	Nucleotide	GBIF	Shared by Nucleotide and GBIF	EID2	GMPD2	BMPO
Bacteria	136	65	55	40	2	58
Viruses	74	13	13	54	9	37
Protozoa	25	9	9	14	5	18
Fungi	54	55	34	—	2	18
Total zoonotic microparasites	289	142	111	108	18	131
Total zoonotic microparasites with geo-information	185	137	97	85	17	129
Host species name	413	—	—	106	45	267
Total associations	1010	—	—	286	67	995
Total associations with geo-information	497	—	—	186	51	977

**Fig. 1:**
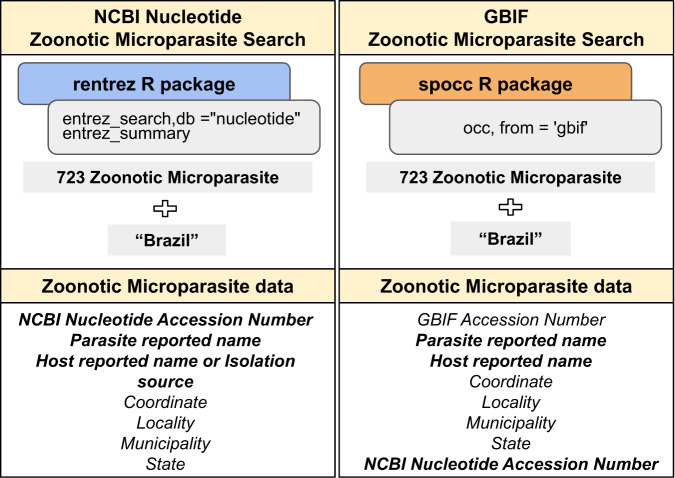
Scheme of the systematic process to automatically retrieve NCBI Nucleotide and GBIF datasets, and the key variables used in this study analyses. A list with zoonotic microparasite names combined with ‘Brazil’ was used in the automated process to retrieve zoonotic microparasite data, using the ‘rentrez’ and ‘spocc’ R packages. Linkable terms between NCBI Nucleotide and GBIF datasets are highlighted in bold.

**Fig. 2:**
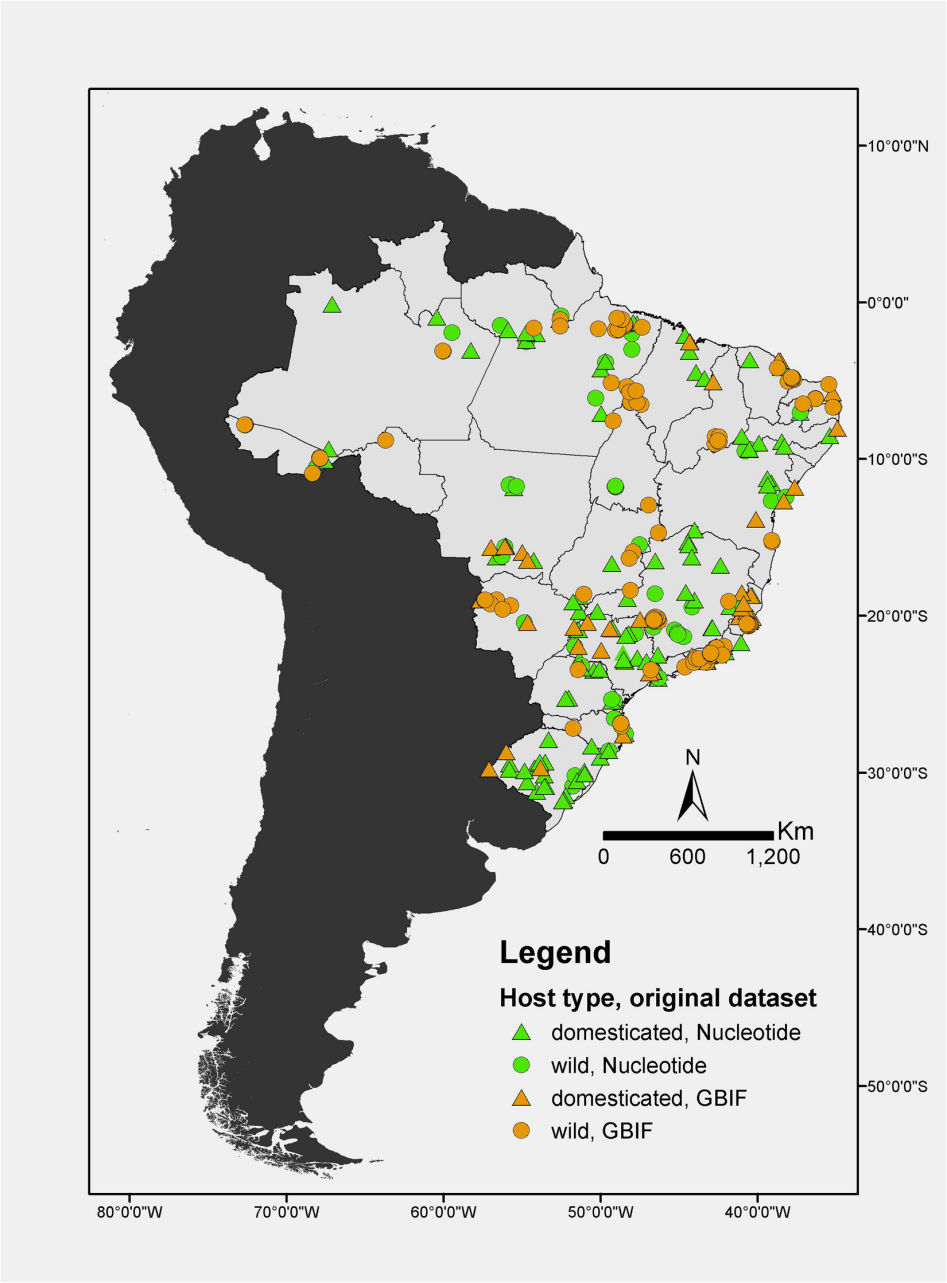
Spatial distribution of georeferenced parasite-host associations from GBIF and NCBI Nucleotide databases. Green symbols denote data from the Nucleotide database, while orange symbols represent data from GBIF. Triangles denote associations between parasites and domesticated animals, whereas circles represent associations between parasites and wild animals.

Interestingly, our automated search failed to retrieve 95 NCBI Nucleotide records (Fig. 1), due to the absence of country specifications. However, we successfully located these records in the GBIF database, where the country of origin ‘Brazil’ was indicated along with the NCBI Nucleotide accession number. Our initial findings reveal that out of the 137 zoonotic microparasites with georeferenced data in GBIF, 40 are absent from NCBI Nucleotide. Similarly, among the 185 zoonotic microparasites with georeferenced data in NCBI Nucleotide, 88 were not found in GBIF. Consequently, our utilisation of georeferenced information marks the first instance of demonstrating the complementarity nature of these two databases (Fig. 2).

### The novel brazilian mammal parasite occurrence data

**Fig. 3:**
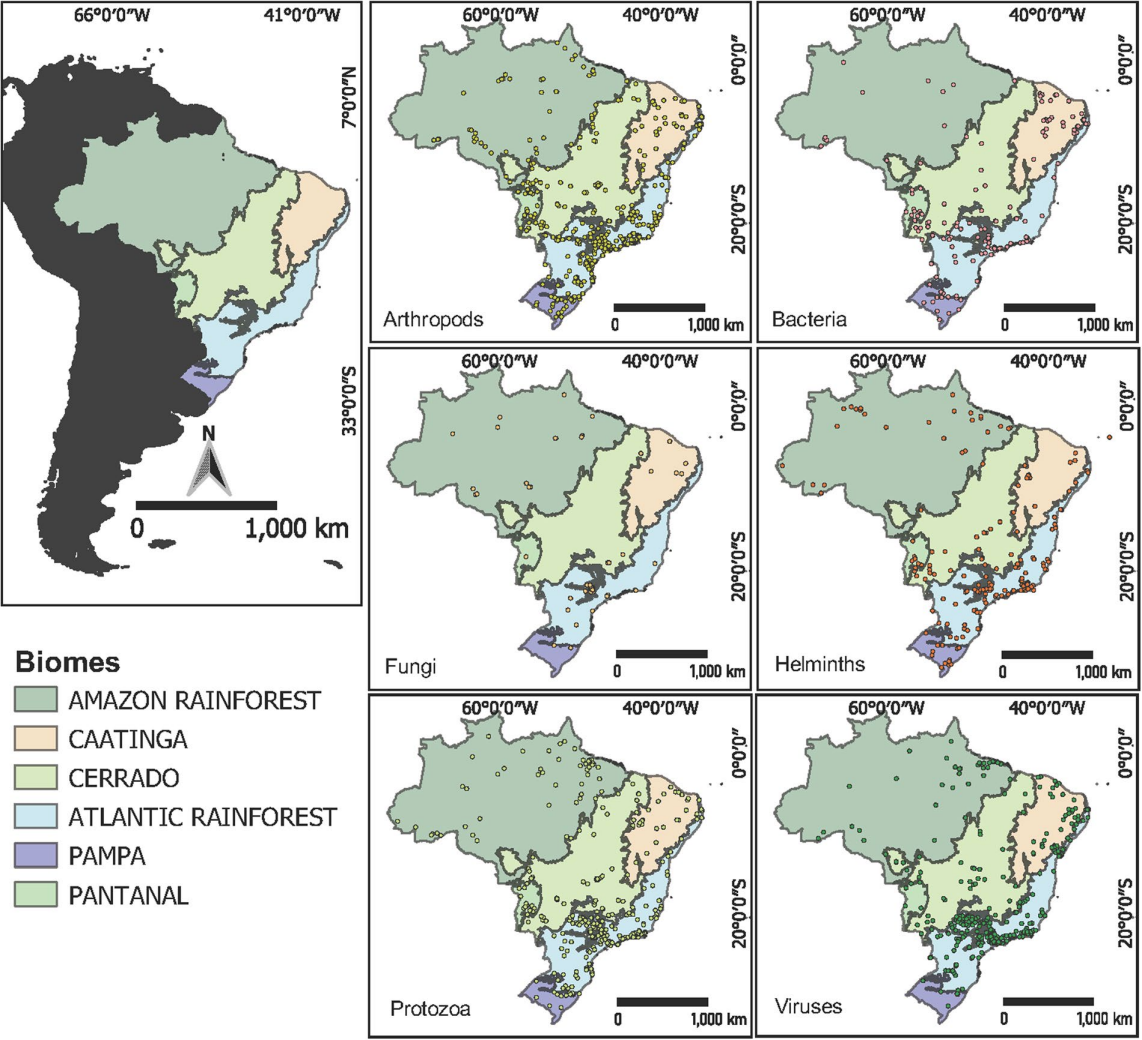
Spatial distribution of georeferenced parasites by taxonomic group across the six Brazilian biomes. The different colors on the Brazilian territory represent the domains of different biomes, while the circles denote the coordinates of occurrence records for different parasite groups.

We compiled a comprehensive dataset of parasites associated with free-ranging native mammal species, encompassing information from 1,121 studies conducted in 719 municipalities across all 27 federative Brazilian units. This dataset, the BMPO, summarises a total of 3,281 associations between parasites (both micro and macro) and free-ranging wild mammal species. Among these associations, 1,025 distinct parasite species were associated with 343 different Brazilian wild mammal species. Specifically focusing on zoonotic microparasites, this dataset includes 131 unique species. We were not able to retrieve even approximate geographic coordinates for 160 records of the total number of parasite-host species associations (N = 3,281). Yet, 125 of these associations included biome information. In total, 3,246 associations provided detailed geographic information beyond the country level, contributing to a more refined understanding of distribution (Fig. 3). Furthermore, the distribution of georeferenced points across the federative unit is concentrated within the Atlantic Rainforest biome, specifically in southeastern Brazil, in the states of São Paulo, Rio de Janeiro, Espírito Santo, and Minas Gerais. This region also accounts for the majority of the studies (488 out of 1,121) that provided either explicit coordinates or enabled retrieval of municipality centroids. In this region, the georeferenced points are mainly associated with the surveillance of rabies and yellow fever viruses identified in bats and primates, the detection of *Rickettsia* spp. in capybaras, and the identification of *Trypanosoma cruzi* and *Toxoplasma gondii* in several other mammals. Additionally, BMPO dataset also encompasses domestic animals, livestock, and species that lack formal recognition by the Brazilian Society of Mastozoology, yet boast confirmed records within the national territory. Although the data concerning parasitic associations with these animals within the BMPO may be somewhat incomplete, we regard it as a promising starting point for future expansion efforts.

When analysing the meta-networks structured by biome, we identified two zoonotic microparasites that exhibit rapid and efficient transmission across different host species. In most biomes, specifically, *Trypanosoma cruzi* and rabies virus had the highest degree centrality, meaning that they interact with a larger number of host species. Furthermore, they showed the highest betweenness centrality, indicating that these microparasites are vital in bridging various segments of the network together. They also had the highest closeness centrality, representing that *T. cruzi* and rabies virus are in close proximity to many other microparasite species concerning the sharing of host species.

Regarding the three calculated network centralities, we observed distinct patterns across biomes. In the Amazon, the most central host species are the tufted capuchin (*Sapajus apella*) with 11 microparasites, the red-handed howler (*Alouatta belzebul*) with seven microparasites, and the insectivorous nine-banded armadillo (*Dasypus novemcinctus*) with six microparasites. In the Atlantic Forest, the capybara (*Hydrochoerus hydrochaeris*), and the bats (*Molossus molossus* and *Artibeus lituratus*) have 18, 14, and 14 microparasites, respectively. In the Caatinga, the rodent *Thrichomys laurentius*, the marsupial *Didelphis albiventris*, and the white-tufted-ear marmoset (*Callithrix jacchus)* have seven, six, and five microparasites, respectively. Whereas in the Cerrado, the maned wolf (*Chrysocyon brachyurus*) has 13 identified microparasites, the rodent *Necromys lasiurus* has eight; and the marsupial *Didelphis albiventris* has six. Within the Pampa biome, canids *Lycalopex gymnocercus* and *Cerdocyon thous* have seven and four microparasites associated, while the primate *Alouatta caraya* has four microparasites. Lastly, in the Pantanal, the most highly connected hosts in the network are *Panthera onca* and Brazilian tapir (*Tapirus terrestris*), each having eight microparasites. Detailed information on the degree, closeness and betweenness centralities of the most central species within each biome is available in Supplementary Table 1.

Our final dataset, the Brazilian Mammal Parasite Occurrence Data, stands as the largest parasite occurrence dataset among mammal orders (as shown in Table 2). BMPO also offers insights into the distribution of these associations across all Brazilian regions. Despite its comprehensiveness, we acknowledge that the data gathered represents just a fraction of the actual ecological reality. This is evident in the steep slope of the parasite accumulation curve depicted in Supplementary Figure S2, suggesting an ongoing increase in identifying parasites in free-ranging wild mammals. Notably, a substantial diversity of parasites remains uncharted. Our dataset comprises not only species associations but also morphological and life-history traits of mammal species, such as body mass, forage strata, activity period, litter size, litter per year, and mode of locomotion. Furthermore, while our dataset may have limited coverage of associations with domesticated and livestock animals, it is worth noting that these categories shared 96 zoonotic microparasites with free-ranging native wild mammals.

**Table 2:** Summary of our new dataset, the Brazilian Mammal Parasite Occurrence Data (BMPO), depicting the number of unique records for each host order taxon (*i.e*., records on parasite species in Brazil). The number of zoonotic parasites is shown in parentheses.

Taxon	Associations	Bacteria	Viruses	Protozoa	Fungi	Helminths	Arthropods
Carnivora	432 (143)	23 (17)	22 (4)	23 (6)	11 (10)	57 (18)	40 (0)
Cetartiodactyla	112 (32)	15 (9)	3 (1)	10 (4)	1 (1)	19 (4)	19 (0)
Chiroptera	1142 (363)	71 (26)	81 (7)	22 (7)	22 (8)	21 (0)	143 (0)
Cingulata	57 (19)	3 (3)	3 (3)	6 (3)	5 (4)	9 (1)	11 (0)
Didelphimorphia	340 (105)	20 (15)	16 (6)	22 (9)	2 (2)	33 (4)	65 (0)
Lagomorpha	2 (1)	—	—	1 (1)	—	—	1 (0)
Perissodactyla	32 (10)	9 (6)	6 (3)	2 (1)	—	—	15 (0)
Pilosa	74 (19)	4 (1)	4 (1)	7 (5)	3 (3)	7 (1)	22 (0)
Primates	238 (145)	23 (17)	28 (22)	15 (9)	4 (3)	19 (0)	10 (0)
Rodentia	852 (229)	38 (24)	57 (15)	22 (10)	3 (3)	108 (10)	137 (0)
Domesticated and Livestock	3688 (366)	152 (74)	73 (30)	57 (15)	33 (25)	106 (32)	44 (0)

## Discussion

### Optimising data utilisation from NCBI Nucleotide and GBIF databases

In this section, we focus exclusively on comparing NCBI Nucleotide and GBIF databases, highlighting how users can enhance the data quality they include in these platforms. We advocate for the combined use of NCBI Nucleotide and GBIF data to quantify potential biases inherent to site selection in studies focused on parasite detection. While such studies often lack geospatial variables, these datasets prove invaluable for addressing global-scale research questions. However, for finer spatial scales, precise geographic coordinates within parasite-host data become essential, particularly when considering factors such as land cover and use. This need is particularly pertinent for megadiverse countries such as Brazil, experiencing fast anthropogenic changes^3^.

DNA sequences comprise a valuable tool in advancing our understanding of species associations^22,23^, NCBI Nucleotide usually offers pathogen sequences and the associated host/source data, which can be exploited for parasite-host association and macroecological studies. Furthermore, NCBI Nucleotide allows the inclusion of sequence data from any study, regardless of publication status. Our findings indicate that the NCBI Nucleotide database contains molecular sequences from a range of zoonotic microparasite-host associations not identified in GBIF, while GBIF contains complementary microparasite records to those in NCBI Nucleotide, facilitating spatial analyses. These databases are linked by the ‘associatedSequences‘ field in GBIF, which corresponds to ‘caption’ in NCBI Nucleotide. The microparasites found in NCBI Nucleotide and GBIF are linked by ‘organism’ and ‘scientificName’ fields in these databases, respectively. Finally, microparasite hosts or isolation sources are identified using ‘associatedTaxa’ in GBIF and ‘isolation source’ or ‘host’ in NCBI Nucleotide. These existing data and metadata fields already demonstrate the level of interoperability between these databases, which could be enhanced through improving data reporting by users.

The most common zoonotic microparasite hosts in the NCBI Nucleotide dataset were domestic species (*i.e*., dogs, cats, cattle, pigs, chickens), probably due to their economic and sociological relevance. However, it’s worth noting that almost all GBIF records linked to the NCBI Nucleotide dataset lack host species identification, although this information is available in the corresponding NCBI Nucleotide record. The records also frequently lack information on the experimental or natural origin of the infection. Thus, scientists including sequences in NCBI Nucleotide and occurrence data in GBIF are feeding these databases with low-precision data. Unfortunately, this practice reduces the overall data quality and limits its usefulness for the research community, particularly in assessing the risks of spillover events. To tackle these issues, it would be advantageous to establish a specific field to indicate whether the deposited data relates to experimentally or naturally infected animals.

We also found that NCBI Nucleotide contains a greater number of unique microparasite-host associations compared to GBIF. Our analysis in both databases focused primarily on zoonotic microparasite-host association data, essential in developing predictive or spillover models. However, we believe that this data may also be useful in ecosystem services studies, such as primary productivity involving plants, algae, and cyanobacteria^24^. Interestingly, some microparasite sequences (11% or 34 sequences) were identified in the primary producers Magnoliopsida and Florideophyceae. Notably, some of the zoonotic microparasites, such as *Klebsiella pneumoniae*^25^ and *Enterobacter asburiae*^26^, influence primary productivity by enhancing plant growth, biomass accumulation and nutrients uptake. Furthermore, it is worth noting that some of these microparasites, including *Escherichia coli*, *Giardia intestinalis*, *Klebsiella pneumoniae*, are transmitted through trophic interactions, making plants an important component of the transmission cycle. The effect of these microparasites on plants is diverse, ranging from endophytic relationships that cause no damage to the hosts, to cases where zoonotic microparasites can alter growth and induce oxidative stress (as seen in Ji *et al*.^27^). Additionally, some of these microparasites are pathogenic for plants, such as the fungus *Fusarium solani* affecting mate (*Ilex paraguariensis*)^28^.

Overall, both the GBIF and NCBI Nucleotide databases, along with their data contributors, exhibit shortcomings in terms of data integration. Users frequently do not properly fill in data fields in repositories, and the repositories themselves lack full interoperability. For example, while GBIF includes a field that links to NCBI Nucleotide (‘associatedSequences’), NCBI Nucleotide lacks a direct field to GBIF, despite GBIF also having an access number (‘gbifID’). Previous studies have already highlighted steps and practices to improve interdisciplinary approach to data collection, data quality, data reuse, reproducibility, and interoperability among biorepositories. These steps include the utilisation of host specimen vouchers or parasite sample vouchers and collection catalogue number in digital databases^29–31^. Remarkably, GBIF presents ‘otherCatalogNumbers’ field, and NCBI Nucleotide includes a ‘specimen voucher’ field, both of which could significantly enhance data traceability if researchers depositing data provide high-precision data, which is frequently available.

### New parasite-host association data synthesis and parasite epidemiological traits: contributions to One Health surveillance

**Fig. 4:**
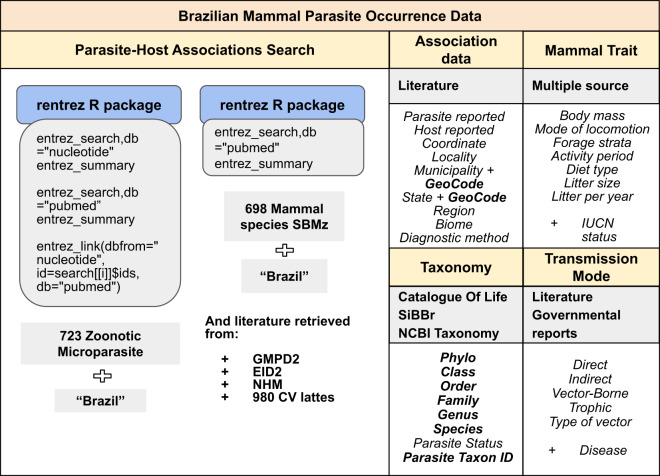
Scheme of the literature source to construct the Brazilian Mammal Parasite Occurrence Data (BMPO) and the associated variables contained in this dataset. Possible linkable terms between BMPO and other biorepositories or Brazilian maps are highlighted in bold. The BMPO construction involved a semi-automated process, including literature extracted from well-established parasite-host databases: Enhanced Infectious Diseases (EID2) database, Global Mammal Parasite v.2.0 (GMPD2), and London Natural History Museum database (NHM), together with the literature used from the Brazilian curriculum vitae platform (Lattes platform). The automated process to retrieve literature was performed using the ‘rentrez’ R package, to access publications associated with NCBI Nucleotide and PubMed databases. Two lists with zoonotic microparasites and Brazilian wild mammal species names were used combined with ‘Brazil’ in the automated step. Mammal traits and parasite transmission modes were gathered from multiple databases, literature, and governmental reports. The scientific nomenclature was updated using Catalogue of Life, the Brazilian Society of Mastozoology list (2020), and NCBI Taxonomy database.

Our study contributes to new developments in parasite-host association data synthesis and highlights their implications for parasite epidemiology, particularly within the One Health framework. To achieve this, we conducted a thorough comparison between the existing databases (NCBI Nucleotide, GBIF, EID2, and GMPD2) and our newly curated BMPO dataset. Our BMPO dataset stands out for its distinct approach, systematically constructed through dedicated research on Brazilian wild mammals. A key highlight is its ability to offer a more refined geographic scale distribution of associations between mammals and parasites, specifically focusing on microparasites and wild mammals (Fig. 4). As a result, the BMPO dataset captures a comprehensive collection of several associations between free-ranging wild mammals and parasites, accompanied by high-quality geographic information tailored to Brazilian taxa. This sets it apart from the global databases previously mentioned.

Although species interactions can be highly variable even on small spatial scales, influenced by environmental factors (e.g., Speer *et al*.^32^), our BMPO dataset incorporates different levels of geographical information, often including centroid coordinate estimates. This dataset is currently the most representative source for spatial parasite-host association data from Brazil. Its fine-grained geographic scale information is invaluable for guiding efforts on parasite and host surveillance. Moreover, it can also be applied to feed predictive models aimed at anticipating the emergence of new host-parasite associations and their potential to disseminate across Brazilian regions. BMPO is also characterised by its user-friendly nature attributed to its relational structure. This architectural framework arranges data into a table with columns and rows, facilitating a clear and intuitive understanding of the interrelationships among different data components.

Assessing host-parasite association via interaction network models and their metrics is crucial for ecological and epidemiological insights. We applied a general meta-network model, using the BMPO database to demonstrate its potential use. By highlighting the most central species (degree, closeness, and betweenness centralities) in Supplementary Table 1 and visualising these associations through meta-networks in each biome, we further extended our understanding of the intricate relationships between host and parasite species. This enables us to delve deeper into the architecture of these relationships within geographically diverse units, even considering heterogeneous land use characteristics. Although certain factors may restrict the geographic dispersion of host species and their associated parasites (*e.g*., D’Bastiani *et al*.^33^), our analyses offer crucial insights. Regarding hosts, network analyses allow us to identify species that facilitate parasite spread. Similarly, from a parasite perspective, we can identify those that present the potential to spread between different mammal species (*e.g*., Wardeh *et al*.^7^, Stella *et al*.^34^, Nieto-Rabiela *et al*.^35^, Albery *et al*.^36^). This approach sheds light on host species that may act as bridges for parasite spillover due to betweenness centrality, such as *Panthera onca* (betweenness = 0.23) in the Pantanal biome.

Our synthesis and analysis here contribute significantly to addressing some interesting questions within Brazilian parasite-host biodiversity: (i) which species parasite wild free-ranging mammals, (ii) which species comprise parasite hosts, (iii) where are the parasites located, and (iv) which parasites are found in a specific region. Furthermore, we provide valuable ecological and epidemiological information that enhances the accuracy of predictive models for zoonosis risk.

### Data limitations and potential enhancements of the established databases and the new dataset

We recognize the challenges involved in documenting all parasite species within a megadiverse and large territorial extent. Such an endeavour is complex due to factors such as the inaccessibility of remote areas, logistical difficulties in detecting parasites at the species level, potential misidentification of host species, and variable research efforts or lack thereof. Especially concerning our interests, we recognize limitations in the ‘associatedTaxa’ field of the GBIF repository. This field often lacks scientific host identification, relying primarily on popular names or even being unavailable, which significantly hampers the study of parasite-host associations. In contrast, the NCBI Nucleotide database offers more comprehensive information, including data associated with deposited sequences (*e.g*., host name, source organism, locality, country), the associated papers, which help retrieve host species, and specific survey locations. Sequences lacking associated publication can still provide spatially distributed species association information, for example. This underlines the potential utility of the NCBI Nucleotide data to retrieve location-related data for parasite-host associations.

Finally, we endorse other calls (*e.g*., Dunnum *et al*.^29^, Thompson *et al*.^30^, Galbreath *et al*.^37^) to promote effective data management practices. We emphasise the importance of enhancing integrated and standardised data storage in biorepositories, bridging genetic and biodiversity databases to advance disease ecology analysis, policy development, and management efforts. Data integration and interoperability are, therefore, major concerns, and FAIR Data Principles (Findable, Accessible, Interoperable and Reusable) are a vital guideline to enhance data reusability for both machines and people in a big data world. One key challenge in dataset integration lies in adopting controlled vocabulary with well-defined term definitions across databases^38^. We encourage the insertion of all types of data in online repositories, recognising that every piece of data holds value to someone.

Specifically for the NCBI Nucleotide and GBIF datasets, several practices could improve data quality: i) informing the host species associated with the studied organism; ii) including taxonomic details, especially in the host field; iii) specifying whether the host was naturally or experimentally infected; and iv) including precise georeferenced data, preferably in the form of coordinates, to enable both temporal and spatial studies. By including these data, we believe depositary researchers can contribute to research effort optimization towards genetic parasite characterization. This also makes data useful for future researchers and interdisciplinary studies, thereby expanding the potential application of the open database. This information is critical in predicting how spatial interactions influence disease dynamics in community assessments, disease ecology, and macroecological studies.

Understanding spatial patterns often faces challenges due to bias in study site locations, a factor that applies to our new dataset (BMPO). We acknowledge a spatially biased distribution of georeferenced points in parasite-host association data, probably stemming from their concentration near rivers and roads where vertebrate sample collection occurs^39^. Moreover, sample sites tend to cluster around major cities, universities, and research institutions. Despite this, it’s important to note that most studies lack precise survey location information. Nevertheless, we have observed that the distribution of host species follows the literature on Brazilian mammal species distribution. For instance, the Atlantic Rainforest harbours the highest bat diversity^40^, while the Caatinga and Cerrado biomes exhibit the highest rodent diversity^41^. Our new dataset, BMPO, represents the best information currently available on parasite-host associations in Brazilian regions. Supplementary Figure S3 illustrates the relative proportion of host species and parasites in each biome, along with zoonotic microparasite transmission modes depicted in Supplementary Figure S4. This dataset holds the potential to enable the scientific community to uncover macroecological patterns and make informed decisions such as prioritising areas for parasite surveillance, monitoring key species (mammal or parasites), and integrating Brazilian environmental datasets into BMPO to improve the accuracy of predictive models.

Moreover, biological interaction data syntheses improve our understanding of hosts and parasite distribution, thus allowing for targeted prevention measures and a One Health surveillance approach. Different ongoing projects, including the Parasite Microbiome Project^42^, Projecting Responses of Ecological Diversity in Changing Terrestrial Systems (PREDICTS)^43^ and the Global Virome Project^44^, are actively working towards this goal. Collaborative studies such as ‘open synthesis’^45^ efforts between empiricist and synthesist communities, alongside refined field collection protocols and organised mammal and parasite archives^37^, are being pursued to optimise and standardise data collection and storage practices.

Overall, we demonstrated herein that the use of different databases crucially contributes to the synthesis of parasite-host knowledge. Notably, we highlight that (i) NCBI Nucleotide data are a significant source of host range information, valuable for predictive models requiring spatially georeferenced association data; and (ii) GBIF data are useful in predictive models requiring spatially georeferenced co-occurrence data. This dual contribution of NCBI Nucleotide and GBIF spatial data are complementary and expand our knowledge of parasite distribution. We suggest that NCBI Nucleotide depositaries should enhance their contribution by including geographic location, while GBIF depositaries should prioritise the inclusion of scientific host names or data sources. Lastly, we present our new dataset, BMPO, which is a result of a rigorous and strong effort to synthesise dispersed data in the scientific literature. This contribution significantly enhances our ability to refine models concerning disease emergence and the distribution of parasite-host associations.

## Methods

### NCBI nucleotide and gbif data

We assessed the NCBI Nucleotide and GBIF databases and their integrated datasets to demonstrate how they can be used as complementary sources in assessing parasite-host associations. We limited our analyses to the zoonotic parasites described in Taylor *et al*.^46^, Jones *et al*.^47^, Olival *et al*.^48^, and Johnson *et al*.^49^. The full list of zoonotic microparasites (bacteria, fungi, protozoa, and viruses) includes 723 microparasites encompassing species, serotype, biotype, serogroup, subspecies, and no rank for the NCBI taxonomic status classification (see Data Availability). We excluded macroparasites (arthropods and helminths), as their identification typically relies on morphological techniques.

To compile data, we retrieved all nucleotide sequences and associated information available in NCBI Nucleotide (https://www.ncbi.nlm.nih.gov/nucleotide/) with Brazil as the origin country up to May 2021. This was achieved through an automated search employing the ‘entrez_search’ function within the ‘rentrez’ package^50^ available in the R platform version 4.0.0^51^. We specified the zoonotic microparasite name and ‘Brazil’ as the search parameter. Similarly, we performed an automated search in the GBIF database using the ‘spocc’ R package version 1.2.0^52^ with identical search criteria. Finally, we used the ‘taxizedb’ R package version 0.3.0^53^ to update species nomenclature.

It is important to note that unlike the new dataset of associations between parasites and wild mammals we present in the following section, the data retrieved from NCBI Nucleotide and GBIF are unrestricted to wild mammals. When analysing NCBI Nucleotide and GBIF data, we aimed to assess the data quality concerning zoonotic microparasite sources (such as the scientific name of the host) and precise geographic locations. We did not incorporate GBIF data into the construction of the new dataset (herein BMPO) due to challenges in tracing the literature source for reported associations. The literature was essential to recover detailed information on hosts, locality, and whether the hosts were free-ranging, since such information was missing from the GBIF platform. Similarly, we did not use NCBI Nucleotide data due to instances where a direct correspondence between the parasite ‘accession number’, the host, and the locality was lacking. Aligning these three elements with information from the literature requires more detailed annotations within NCBI Nucleotide.

### New parasite-host association dataset, mammals and parasite traits: brazilian mammal parasite occurrence data

Initially, we aimed to compile comprehensive data on the occurrence of associations between parasites and wild mammals in Brazilian territory, drawing from well-established databases: the Enhanced Infectious Diseases database (EID2; Wardeh *et al*.^20^), the Global Mammal Parasite Database v.2.0 (GMPD2; Stephens *et al*.^21^), and the host–helminth parasite occurrence records from the London Natural History Museum database (NHM)^54^. However, as we gathered information from these databases, we noticed that the geographical distribution of parasite-host associations did not align with the existing literature. Data on parasite-host associations in these databases referred to specific literature sources. Therefore, we cross-referenced this literature to complete the locality information and certify that the hosts were indeed free-living individuals in their natural habitats. We also supplemented this data by incorporating additional literature, thus creating an extensive dataset encompassing native free-ranging mammal species and their associated parasites across Brazil.

We collected EID2 data using the ‘rvest’ version 0.3.6^55^, and accessed NHM records using the ‘helminthR’ package version 0^56^. Employing a semi-automatically approach, we conducted cross-searches on the PubMed and NCBI Nucleotide databases for a list of native Brazilian mammal species provided by the Brazilian Society of Mastozoology in 2020^57^ through the ‘rentrez’ package (see Data Availability). Similarly, we conducted these searches for the same list of zoonotic microparasites used in the NCBI Nucleotide and GBIF queries. We employed a combined search of mammal species names and ‘Brazil’ within the title field, applying the same combination for zoonotic microparasite names. For instance, ‘*Cerdocyon thous’* AND ‘Brazil’, ‘yellow fever virus’ AND ‘Brazil’, and so forth, one by one. Given the semi-automated nature of this extensive search, we restricted the search to the title field. Viruses, bacteria, protozoa, fungi, helminths, and ectoparasites (arthropods) are included in this new dataset. We also conducted an additional search on the Brazilian curriculum vitae platform (lattes.cnpq.br, May 2021), retrieving papers authored by 980 researchers. The list of primary papers used for author retrieval is available in Data Availability. Although it was not the primary aim of our study, we also retrieved associations involving domesticated animals, livestock, and associations with arthropod and helminth parasites. These associations were already available in the literature used by EID2, GMPD2, NHM, and literature resulted from our semi-automated search, which uncovered such associations when searching for the wild mammal species and ‘Brazil’ in the title field.

From each selected paper, we extracted the following information: host (species level), parasite (from all taxonomic levels), parasite detection method, the most detailed spatial scale (*i.e*., geographic coordinates, municipalities, or states), title, author(s) and publication year. We manually excluded case reports involving humans, experimental inoculation studies, papers derived from raw milk or raw meat analyses, and parasite associations with captive hosts. Species taxonomy followed the Brazilian Society of Mastozoology 2020^57^. Synonymy of mammal, arthropod, and helminth species was aligned with the Catalogue of Life website (https://www.catalogueoflife.org) and the Brazilian Fauna Taxonomic Catalog (https://www.sibbr.gov.br). We used the NCBI Taxonomy database (https://www.ncbi.nlm.nih.gov/taxonomy) for the other parasite groups.

Additionally, we retrieved morphological and life-history data^58–81^ for wild mammal species and zoonotic microparasite transmission modes^82–89^. We restricted the research on the transmission modes to zoonotic microparasites, since helminths usually have a complex and still poorly-known life cycle.

### Structuring and technical validation of the BMPO

As previously described, gathering of host and parasite information from different sources required a significant effort to ensure the reliability and coherence of the final database we generated. This undertaking also encompassed the challenge of refining the vocabulary employed within the BMPO dataset. We used a controlled vocabulary related to taxonomy (species, genus, order, class, and Phylo), geographical descriptors (locality, municipality, state, region, and biome), and the diagnostic method. Our taxonomy nomenclature adhered to the aforementioned institutions, while geographic names followed IBGE (Brazilian Institute of Geography and Statistics) guidelines for municipalities, states, regions, and biomes. We rectified minor typographical errors in geographic names, and in the ‘locality’ field, we standardised at least the names of preservation areas and farms. Furthermore, for the ‘DiagnosticMethodParasite’ vocabulary, we established classes for the reported methods and documented them in the metadata file (see Data Availability).

Beyond the creation of the accessible final database, we hold an interest in its continual enhancement, either by the inclusion of new relationships or by expanding the potential analyses of the existing dataset. In the future, BMPO updates will be on an institutional platform with an interface that users can include new records. Consequently, it is imperative for the data to be structured comprehensively, capable of articulating pertinent relationships and reinforcing the cohesive structures of the collected, stored, and scrutinised information. This need for a formal data model and its effective management becomes even more evident when handling substantial volumes of data, rendering a purely manual assessment impractical. As data quantities grow, automation tools and data models that accurately reflect the research context are paramount for data synthesis and analysis. Biodiversity informatics, a cross-disciplinary field, exemplifies the growing importance of comprehending and refining biodiversity information, encompassing its representation to its integration by different sources^90^.

**Fig. 5:**
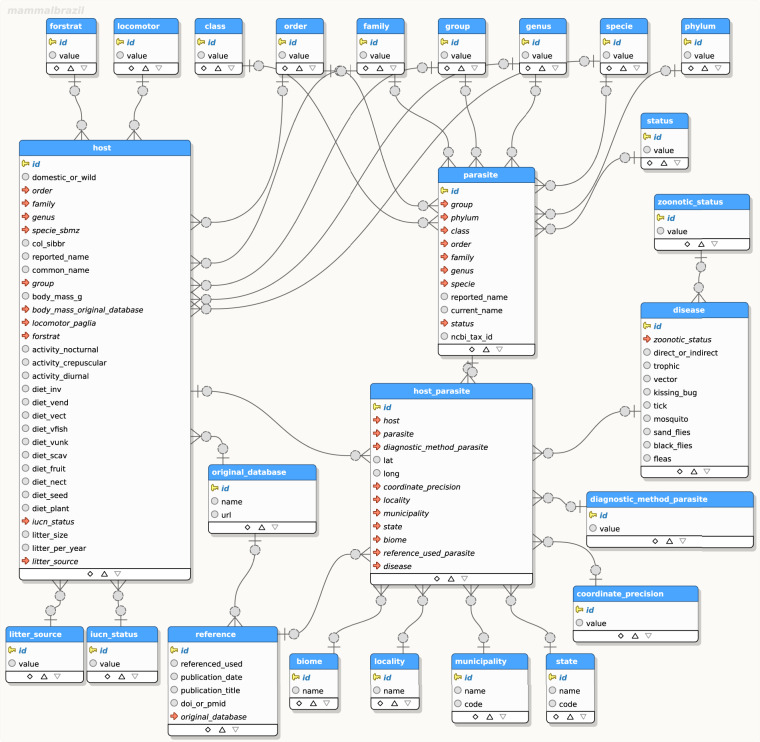
Data model designed to manage the Brazilian Mammal Parasite Occurrence Data (BMPO). The model was implemented in PostgreSQL. The parasite-host relationship is the central unit of the entire model and subsequent analysis. The model is structured into five subset: (i) users can search for host ecological data, (ii) publication relating to parasite-host association, (iii) location of occurrence of host-parasite associations, (iv) parasites that circulate in wild mammals, and (v) zoonotic microparasite epidemiological data.

In this study, beyond the retrieval, conversion, filtering, and processing phases (via automated routines executed in R), we established a new database model to represent the final context of analysis. This framework facilitated data management and inconsistency identification. Coupled with the database model, a data import process was established using the Python programming language to integrate the data into the PostgreSQL model. Once the database model was established and fully managed, any inconsistencies were rectified or eliminated before exporting the dataset to the final data repository. For example, geographic coordinates underwent thorough validation since some studies provide erroneous coordinates, often outside Brazilian territory. In most cases, geographical coordinates represent an approximation of the centroid reported in decimal degrees (Datum WGS84) using coordinates provided by papers, and Google Maps. While these coordinates were frequently imprecise, they still provide an approximation of the study location. Ultimately, the detailed data model implementation is presented in Fig. 5, where the parasite-host relationship is the central unit of the entire model and subsequent analysis.

### Data analysis

#### Mapping parasite-host association distributions

We extracted the geographical coordinates of each record and described them as latitude and longitude (decimal degrees, WGS84 Datum). When not available, we used the approximate location from study area descriptions using Google Maps whenever possible. When no detailed information was available, we included the centroid of the smallest available administrative area (e.g., municipality, state). Further, we categorised the coordinates according to their source, as informed by the authors: locality, municipality, or state centroid. To depict the spatial distribution of records across Brazilian regions we used the QGIS software version 3.12^91^.

#### Meta-network analyses

To create a binary interaction meta-network for each Brazilian biome, we employed presence (1) or absence (0) of a pairwise interaction between a host and a zoonotic microparasite. To understand the contribution of each species (nodes) to network topology (i.e., species role), we calculated key centrality metrics using the BMPO dataset. Specifically, we calculated degree centrality, which comprises the number of interactions of a given species; closeness centrality, which is the sum of the number of shortest distances (number of interactions) between species *i* and all other species in the network; and betweenness centrality, which consists in the sum of the number of pairs of species whose shortest path lengths are connected through species *i*.

### Supplementary information

Supplementary Figures

Supplementary Table

## Data Availability

The datasets are systematically organised as a set of interconnected *.xlsx tables and *.RData, linked by different taxonomic units of parasite and host names. These designations can be linked to publicly available repositories housing scientific names for parasites and mammals. Geographical identifiers, represented by 7-digit municipality codes and 2-digit state codes, are associable with Brazilian maps in shapefile format, and other harmonised datasets containing geographical information. All data, metadata table, and data source references pertaining to the BMPO dataset have been deposited within the figshare repository^92^. This repository includes the following components: (i) zoonotic microparasite list, (ii), Brazilian wild mammal species list, (iii) primary papers list, (iv) Nucleotide data, (v) GBIF data, (vi) BMPO data, and (vii) BMPO metadata. Please refer to the readme file for a more detailed description of the files.

## Code availability

For data processing and generation of data products, we relied on open-source software packages. Specific software versions, when relevant, have been duly specified. The R code used to retrieve data from NCBI Nucleotide, GBIF, EID2, and NHM datasets is available at figshare^92^. Codes for cross-referencing Nucleotide and PubMed, as well as meta-network analysis, are housed in the same repository. GBIF and NCBI Nucleotide data are within the *.RData file, which can be loaded into R using the ‘load’ statement.
