# Supplementary material for: Integrating databases for spatial analysis of parasite-host associations and the novel Brazilian dataset
Source: Sci Data. 2023 Nov 2;10:757. doi: 10.1038/s41597-023-02636-8 (PMC10622529; doi:10.1038/s41597-023-02636-8)
Supplement: Supplementary file 1 — Supplementary Figures [file 41597_2023_2636_MOESM1_ESM.pdf]

Integrating databases for spatial analysis of parasite-host associations and the novel Brazilian dataset

Gabriella L. T. Cruz, Gisele R. Winck, Paulo S. D'Andréa, Eduardo Krempser, Mariana M. Vidal, Cecilia S. Andreazzi

Supplementary Information

This supplement contains figures that describe the NCBI Nucleotide, GBIF, and Brazilian Mammal Parasite Occurrence Data (BMPO) datasets.

Table of contents

Figure S1 Proportion of data with host/source information and data with geographic coordinates in NCBI Nucleotide and GBIF dataset…..……..………………………………2

Figure S2 Literature data from the BMPO**.** Number of published papers (columns) reporting parasites and the accumulated number of parasites (points) in free-ranging wild native mammals in Brazil between 1945 and 2020. (a) Micro and macroparasites accumulation curve and (b) zoonotic microparasites accumulation curve…………….3

Figure S3 Host and parasite species data from the BMPO. Graphics showing the relative proportion of (a) host species by taxonomic group and (b) parasite species by host order recorded for each biome……………………………………………………..4

Figure S4 Transmission mode data from the BMPO. Number of zoonotic microparasites by taxonomic group and transmission mode. VB = Vector Borne, T = Trophic, DC = Direct Contact, and ID = Indirect Contact…………………………………………………………………………..……5

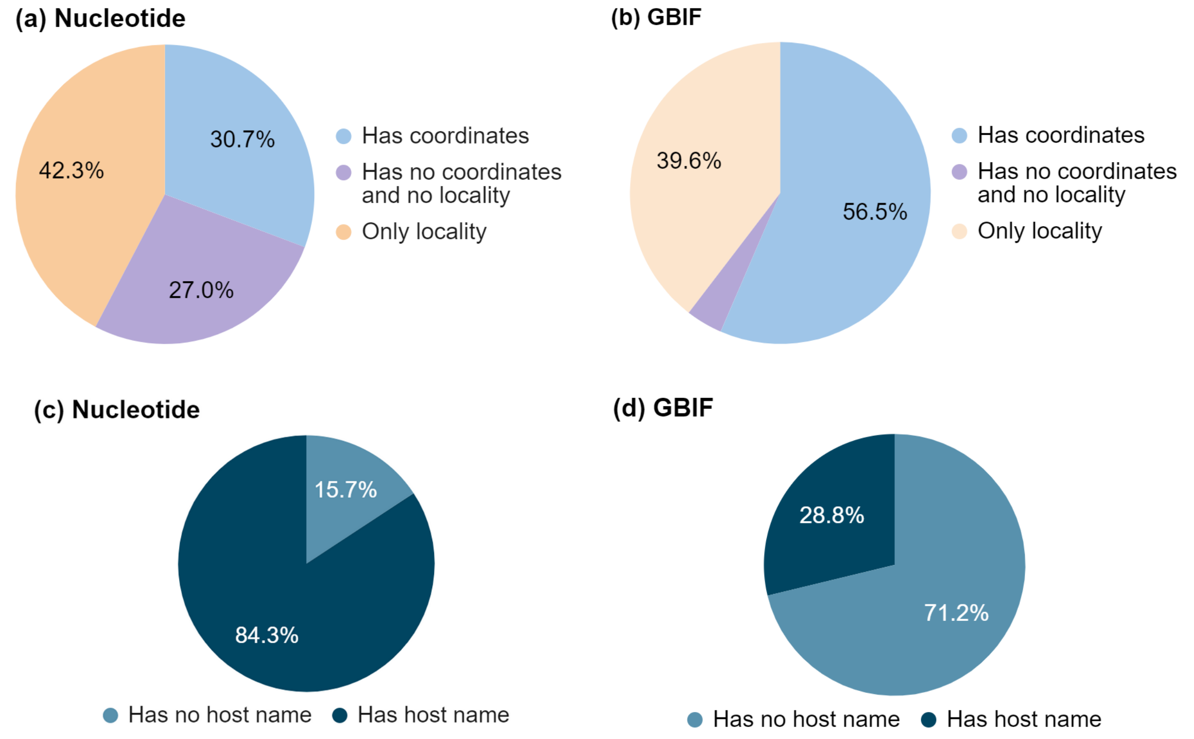

Figure S1 Proportion of data with host/source information and data with geographic coordinates in NCBI Nucleotide and GBIF dataset.

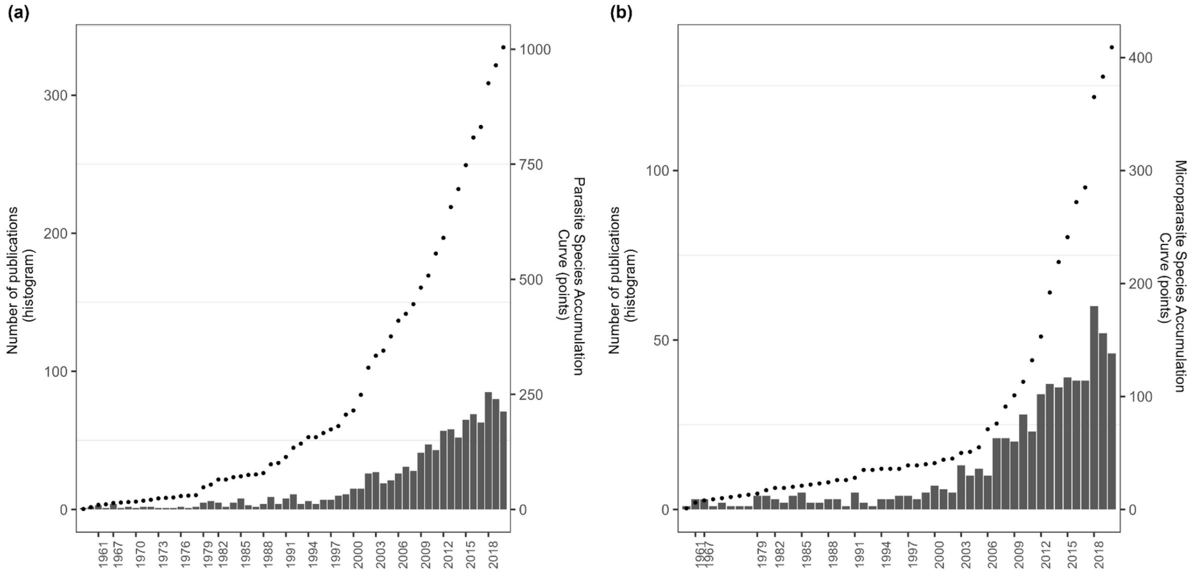

Figure S2 Literature data from the BMPO**.** Number of published papers (columns) reporting parasites and the accumulated number of parasites (points) in free-ranging wild native mammals in Brazil between 1945 and 2020. (a) Micro and macroparasites accumulation curve and (b) zoonotic microparasites accumulation curve.

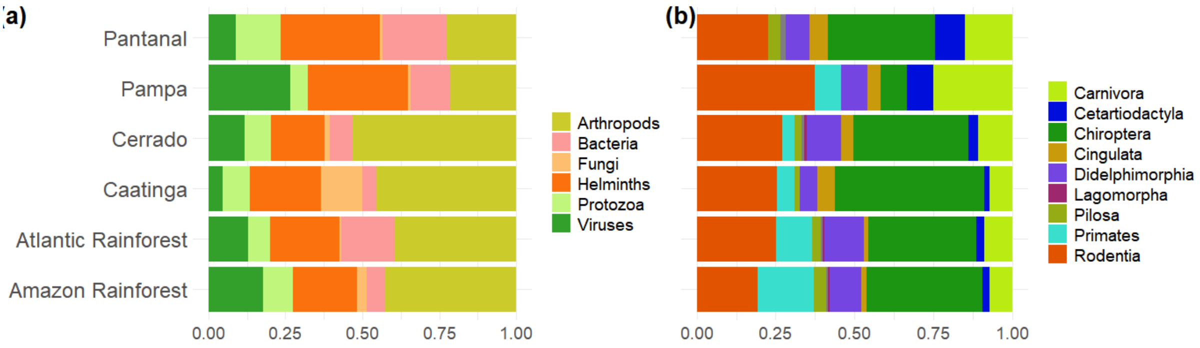

Figure S3 Host and parasite species data from the BMPO. Graphics showing the relative proportion of (a) host species by taxonomic group and (b) parasite species by host order recorded for each biome.

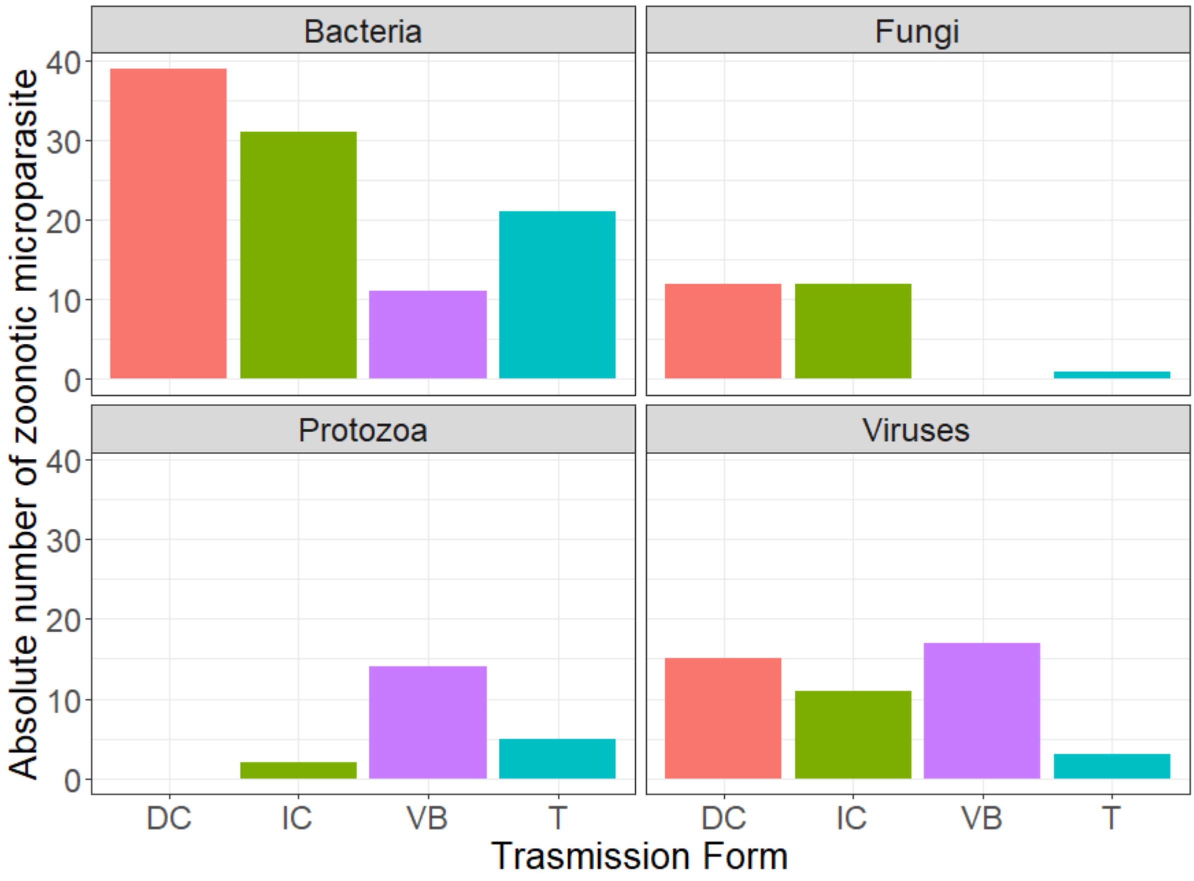

Figure S4 Transmission mode data from the BMPO. Number of zoonotic microparasites by taxonomic group and transmission mode. VB = Vector Borne, T = Trophic, DC = Direct Contact, and ID = Indirect Contact.
